# Degradation of vicine, convicine and their aglycones during fermentation of faba bean flour

**DOI:** 10.1038/srep32452

**Published:** 2016-08-31

**Authors:** Carlo Giuseppe Rizzello, Ilario Losito, Laura Facchini, Kati Katina, Francesco Palmisano, Marco Gobbetti, Rossana Coda

**Affiliations:** 1 University of Bari “Aldo Moro”, Department of Soil, Plant, and Food Science, Via Amendola 165/a, 70125 Bari, Italy; 2 University of Bari “Aldo Moro”, Department of Chemistry, Via E. Orabona 4, 70125 Bari, Italy; 3 University of Bari “Aldo Moro”, SMART Inter-department Research Center, Via E. Orabona 4, 70125 Bari, Italy; 4 University of Helsinki, Department of Food and Environmental Sciences, Agnes Sjioberginkatu 2, Helsinki, Finland

## Abstract

In spite of its positive repercussions on nutrition and environment, faba bean still remains an underutilized crop due to the presence of some undesired compounds. The pyrimidine glycosides vicine and convicine are precursors of the aglycones divicine and isouramil, the main factors of favism, a genetic condition which may lead to severe hemolysis after faba bean ingestion. The reduction of vicine and convicine has been targeted in several studies but little is known about their degradation. In this study, the hydrolysis kinetics of vicine and convicine and their derivatives during fermentation with *L. plantarum* DPPMAB24W was investigated. In particular, a specific HPLC method coupled to ESI-MS and MS/MS analysis, including the evaluation procedure of the results, was set up as the analytical approach to monitor the compounds. The degradation of the pyrimidine glycosides in the fermented flour was complete after 48 h of incubation and the aglycone derivatives could not be detected in any of the samples. The toxicity of the fermented faba bean was established through *ex-vivo* assays on human blood, confirming the experimental findings. Results indicate that mild and cost effective bioprocessing techniques can be applied to detoxify faba bean also for industrial applications.

Faba bean (*Vicia faba* L.) is a leguminous plant belonging to the *Fabaceae* family, able to grow in different climates^1^. Faba bean is an important plant for human consumption, having a valuable nutritional composition, particularly rich in high quality protein^2^, and providing a balanced diet of lysine-rich protein, carbohydrates, fibre, and phytochemicals^3^. In addition, faba bean is suitable to replace soy bean in the feed ration of different animals and can promote more self-sufficiency of European countries in the import of plant proteins^3^,^4^.

In spite of these advantages, faba bean, like the rest of legumes, contains compounds showing an “anti-nutritional” effect, i.e., having the potential to cause adverse effect on nutrition. Collectively described as anti-nutritional factors, these compounds impair the above described benefits when included in the daily diet^5^. Among these, phytic acid, saponins, lectins, alkaloids and others reduce the digestibility of seeds and may lead to some pathologic conditions^6^. In particular, faba beans are rich in two glucosidic aminopyrimidine derivatives, vicine and convicine, which, upon hydrolysis of the β-glucosidic bond between glucose and the hydroxyl group at C-5 on the pyrimidine ring, generate the aglycones divicine (2,6-diamino-4,5-dihydroxypyrimidine) and isouramil (6-amino-2,4,5-trihydroxypyrimidine), respectively^2^. The molecular structures of the four compounds (including the two possible tautomeric forms of each aglycone) are reported in Fig. 1. Divicine and isouramil have been identified as the main factors of favism, a life-threatening hemolytic crisis that can result from the ingestion of faba beans by susceptible individuals, having low-activity variants of erythrocytic glucose 6-phosphate dehydrogenase (G6PD)^7^. Since G6PD regulates the production of NADPH in the red blood cell by the hexose monophosphate shunt, individuals affected by G6PD deficiency are unable to regenerate reduced glutathione and are undefended against oxidative stress. As result, G6PD deficiency accelerates normal senescence and enhances the precocious removal of chronologically young, yet biologically old, cells^8^. In this sense, it has also been clarified that the term hemolytic anemia is misleading, as red blood cells do not lyse but are removed by phagocytosis^8^. Acute hemolysis caused by faba bean ingestion in G6PD deficient individuals (favism) is described as being the best studied natural model of oxidative damage^8^.

Vicine and convicine are mostly found in the seeds, where they reach concentrations up to 5 mg and 2 mg/g of dry weight, respectively^9^. Faba bean seeds possess β-glucosidase activity varying according to different growth stage: it is very low in young seeds, reaches a maximum in ripe seeds and drops again in older seeds^10^. The enzyme is potentially inactivated during faba bean processing, such as cooking and seed drying, but divicine and isouramil can still be produced by means of microbial β-glucosidases during digestion in the large intestine and cecum^11^. Hence, favism can be induced by the intake of both raw and cooked faba beans. The reduction of vicine and convicine was addressed in several studies, since their presence has limited the use of faba bean as both food and feed^12^. Cultivars with reduced vicine and convicine contents have been selected, but are not commercially available for every climatic condition and reduction to zero of these compounds has not been achieved yet^2^,^13^. Different processing methods (air classification, roasting, boiling) have achieved a reduction of vicine and convicine, for example, heat treatment can impact on the content of the glycosides, partially thermolabile, with convicine being the most heat sensitive^14^. Harsher treatments, such as continuous flow soaking in tap water or in acid, alkaline or neutral aqueous media, are effective in removing vicine and convicine from faba bean seeds and flour, but at the same time can negatively impact on other important properties^15^,^16^. Some studies focused on bioprocessing methods aiming at the selective destruction of the two β-glucosides. For instance, the addition of almonds as a source of β-glucosidase to faba bean paste reduced the concentration of vicine and convicine during a short incubation time in acidic environment^9^. In the same context it was observed that divicine and isouramil concentration decreased after the treatment, hypothesizing their inactivation in the processed faba bean paste^9^. Fungal β-glucosidase from *Aspergillus oryzae* and *Fusarium graminearum* and from lactic acid bacteria effectively degraded the pyrimidine glycosides from faba bean suspension and flour, respectively^17^,^18^. In detail, *Lactobacillus plantarum* showing β-glucosidase activity was used as starter to ferment faba bean flour, achieving a reduction of vicine and convicine of more than 90% and positive impact on the content of other anti-nutritional compounds^18^. Despite all these information on vicine and convicine degradation, very little is still known about the fate and, in particular, about the abundance and the toxicity of the favism-inducing derivatives, divicine and isouramil, in the processed faba bean.

In this study we investigated, through the set-up of a specific liquid chromatography-mass spectrometry (LC-MS) method, the hydrolysis kinetics of the pyrimidine glycoside vicine and convicine and of their derivatives during fermentation with selected strain of *L. plantarum*. The toxicity of divicine and isouramil in the fermented faba bean was finally established through *ex-vivo* assays on human blood.

## Results

### Faba bean flour fermentation

The faba bean flour had a moisture content of 9.45 ± 0.72%. The protein concentration was 35.7 ± 1.2% of dry matter (d.m.), while lipids were 1.63 ± 0.25% of d.m. The concentration of dietary fibers and ash resulted 7.23 ± 0.75% and 3.87 ± 0.12% of d.m., respectively. Carbohydrates were 51.2 ± 2.1% of d.m. Faba bean flour microbial composition was characterized by total mesophilic aerobic bacteria (cell density of 4.01 log cfu/g), presumptive lactic acid bacteria (5.74 log cfu/g) and low amount of enterobacteria (<2.0 log cfu/g). Yeasts and molds in low density (1.15 and 2.0 log cfu/g, respectively) were also found.

Compared with the beginning, the cell density of lactic acid bacteria increased significantly (*P* < 0.05) during incubation in both FF and spontaneously fermented control (Ct) of ca. 2 and 1 log cycles, respectively (see Table 1 and Fig. 2). A slight growth was observed for yeasts (Table 1), while the cell density of the other microbial groups (total mesophilic aerobic bacteria and enterobacteria) did not change (*P* > *0.05*) in any of the doughs (data not shown).

The chemically acidified control A-Ct containing antibiotics was characterized, before fermentation, by pH and TTA values of 4.52 and 15.51 mL mL NaOH 1 N, respectively, and slight but not significant (*P* < 0.05) changes were found throughout the incubation (Table 1). The pH and TTA of the other doughs before incubation were 6.45 and 6.02 mL NaOH 1 N, respectively. After incubation, pH decreased significantly (*P* < 0.05) in FF and Ct (see Table 1 and Fig. 2). As expected, the highest decrease was shown for the controlled fermentation (FF) and an opposite trend was shown by TTA (Table 1). Relevant concentrations of lactic and acetic acid were found in FF (Table 1) and significantly (*P* < 0.05) lower amounts were found in Ct.

The β-glucosidase activity of faba bean flour corresponded to 0.048 ± 0.014 U. After dough mixing and 48 h of incubation, the activity in the water soluble extracts obtained from the doughs corresponded to 0.198 ± 0.012 U for FF, while it was 74 and 80% lower in Ct and A-Ct, respectively (Table 1).

### Preliminary LC-MS investigation on vicine, convicine and their aglycones

HPLC followed by ESI-MS and MS/MS analysis was adopted as the analytical approach to monitor vicine, convicine and their aglycones in FF, Ct and A-Ct. Before proceeding with the extracts obtained from doughs, the commercial standard of vicine was analyzed by direct infusion-ESI-MS and MS/MS. In particular, MS/MS was used to reconstruct the vicine fragmentation pattern, that could be useful for its unequivocal identification in faba bean flour doughs. In this case, a unique signal, located at a *m/z* ratio 143.1, was observed in the MS/MS spectrum obtained by isolating and fragmenting, in the 3D-ion trap of the LCQ spectrometer, the main isotopologue of the vicine [M+H]^+^ ion, i.e. using the *m/z* isolation window 305.3 ± 0.5. Since the signal at *m/z* 143.1 corresponds to the main isotopologue of the [M+H]^+^ ion of divicine, the only possible fragmentation of vicine, at least under the low energy CID conditions achievable with the LCQ 3D-ion trap, consists in the detachment of its glycosylic moiety (mediated by a 1,3-H transfer from the latter to the O atom linked to carbon 5 of the divicine ring). It is important to point out that the [M+H]^+^ ion of divicine was generated from the [M+H]^+^ ion of standard vicine already during the ESI process and/or during the transfer of this ion towards the 3D-ion trap, since a signal at *m/z* 143.1 was detected (with a relative abundance of ca. 20%, certainly a not negligible value) also in the ESI-MS spectrum of vicine. In a subsequent stage, the extract obtained from the faba bean flour^19^ was analyzed. As shown in Fig. 3, the elution of vicine and convicine could be monitored both by UV detection at 280 nm and by MS detection, performed in a serial configuration (this is the reason for the 0.06 min shift apparently observed in Fig. 3 between the retention times of the same compound in the UV and MS chromatograms). In the case of MS detection, eXtracted Ion Current (XIC) chromatograms, i.e. traces resulting from the display of the ion current measured for a specific *m/z* interval vs. retention time, could be obtained for vicine and convicine.

The second isocratic step of the elution gradient was purposely extended far beyond the elution of vicine and convicine (i.e., until 14 min), since this enabled an improvement of the separation between them and other polar compounds contained in the flour extract, whose peaks were clearly detected in the LC-ESI-MS Total Ion Current (TIC) chromatogram obtained for the flour extract, also shown in Fig. 3. The additional signal observed in Fig. 3 for the XIC trace at *m/z* 306.3, perfectly aligned to the peak related to vicine in the XIC trace at *m/z* 305.3, is due to the M+1 isotopologues of vicine [M+H]^+^ ion, i.e. to ions including a 1 Da heavier isotope for one of the atomic constituents of the vicine molecular structure. As for MS/MS analysis, when the convicine [M+H]^+^ ion was isolated and fragmented in the 3D-ion trap during the LC-MS run a unique ion was detected, at *m/z* 144.1, corresponding to the [M+H]^+^ form of isouramil. This finding indicates that the fragmentation pathway of convicine (detachment of the glycosylic moiety, with generation of the protonated aglycone) is identical to that of vicine, which is not surprising, due to the high structural similarity between vicine and convicine.

In view of the possibility that the aglycones of vicine and convicine could be present in the faba bean flour extracts during and after fermentation, the evaluation of their chromatographic and mass spectrometric behavior, under the same conditions adopted for vicine and convicine analysis, was accomplished before proceeding to real samples analyses. In particular, an experiment based on the β-glucosidase-catalyzed hydrolysis of standard vicine to divicine, described in detail in Section S2 of the Supplementary Information, provided an approach to distinguish divicine eventually generated during faba bean flour fermentation from that arising from the gas phase spontaneous fragmentation of vicine during ESI-MS analyses; a similar approach was then adopted, by analogy, in the case of isouramil (see next paragraph and Section S2 and Figure 1S in the Supplementary Information).

### LC-ESI-MS analyses of extracts obtained from fermented faba bean flour doughs

As explained before, aliquots of FF, Ct, and A-Ct were withdrawn before and after 2, 12, 24 and 48 h of incubation and the corresponding acid extracts were analyzed by LC-ESI-MS. XIC chromatograms were retrieved after each analysis for the *m/z* ratios related to vicine, convicine and their aglycones (305.3, 306.3, 143.1 and 144.1). In spite of the presumably higher complexity of incubated doughs, no interference from isobaric compounds was found in the four XIC traces (as it was observed in the faba bean flour extract itself, see Fig. 3). Indeed, as shown through selected examples, in Figures 2S and 3S of the Supplementary Information, although their intensity was generally found to decrease, even remarkably, with fermentation time, peaks located at the retention times of vicine and convicine were clearly detected in the XIC traces obtained for the *m/z* ratios 305.3 and 306.3, respectively. MS/MS acquisitions targeted on these ions (data not shown) confirmed unequivocally that they were related to vicine and convicine still present in the flour extracts.

**Figure 1:**
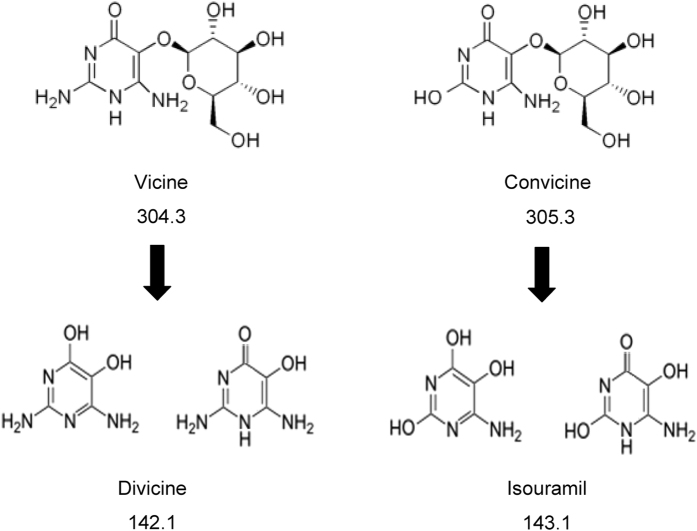
Molecular structures of vicine, convicine and their respective aglycones, divicine and isouramil. The two possible tautomeric forms of the aglycones are reported. Monoisotopic molecular weights, rounded off to the first decimal figure (consistently with the available mass resolution/accuracy) and referred to the main isotopologues of the four compounds in their neutral form, are also indicated.

**Figure 2:**
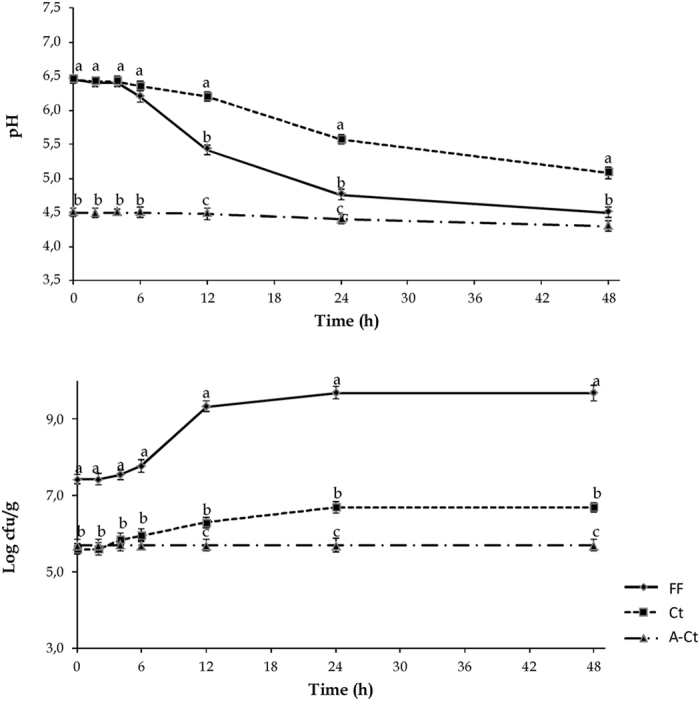
pH and presumptive lactic acid bacteria cell density of faba bean doughs incubated at 30 °C for 48 h. FF, fermented dough, inoculated with *Lactobacillus plantarum* DPPMAB24W (7.30 log cfu/g initial cell density); Ct, spontaneously fermented control dough; A-Ct, not inoculated and chemically acidified dough, added with antibiotics (0.1% wt/vol, cycloheximide, and 0.1% wt/vol chloramphenicol). The data are the means of three independent experiments. ^a–c^Values with different superscript letters differ significantly (*P* < *0.05*).

**Figure 3:**
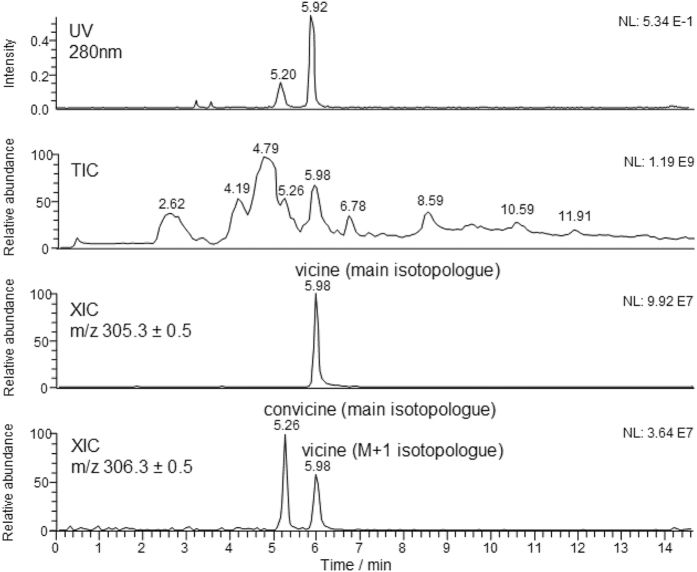
Comparison between the detailed views (0–14 min interval) of the UV chromatogram (λ = 280 nm), the ESI-MS Total Ion Chromatogram (TIC) and the ESI-MS eXtracted Ion Current (XIC) chromatograms, obtained for the main isotopologues of the [M+H]^+^ ions of vicine (*m/z* 305.3, retention time: 5.92 min for UV detection and 5.98 min for MS detection; the difference is due to the fact that MS detection was performed after UV detection, in a serial configuration) and convicine (*m/z* 306.3, retention time: 5.20 min for UV detection and 5.98 min for MS detection), referred to the LC-UV/ESI-MS separation of the acid extract of faba bean flour. Note that the peak detected at 5.98 min in the XIC trace retrieved for the *m/z* ratio 306.3 is due to the M+1 isotopologues of the [M+H]^+^ ion of vicine (i.e. ions whose structures contains a 1 Da heavier isotope for one of the constituting atoms).

As for vicine/convicine aglycones, a peak was observed in each of the XIC traces obtained for the *m/z* ratios 143.1 (divicine) and 144.1 (isouramil) and was always well aligned with the one related to the *m/z* ratios 305.3 and 306.3, respectively (see Figures 2S and 3S for some examples). However, none of those peaks exhibited the peculiar broadening observed in the XIC trace relevant to the *m/z* ratio 143.1 when divicine generated upon enzymatic hydrolysis of vicine had been analyzed (see Fig. 1Sb). Such broadening was tentatively explained with the generation (in solution) of the two different tautomers of divicine (see Fig. 1), likely experiencing a slightly different interaction with the C18 stationary phase and thus exhibiting a difference in retention time. By analogy, a similar effect was hypothesized for isouramil eventually generated in solution from convicine. Interestingly, the effect was not observed in the XIC traces referred to the ions at *m/z* 143.1 and 144.1 when these were certainly related only to divicine and isouramil generated through spontaneous gas-phase fragmentation of vicine and convicine, respectively (see, for example, Figure 1Sa). It is then likely that the tautomerism leading to the two different forms of divicine and isouramil shown in Fig. 1 occurs easily in solution but is hindered in the gas phase.

Starting from these considerations, the absence of peak broadening in all the XIC traces obtained for the *m/z* ratios 143.1 and 144.1 referred to extracts of fermented faba bean flour led us to hypothesize that vicine and convicine aglycones never reached significant concentrations during the fermentation process (likely due to a fast degradation after their formation). The peaks detected in those XIC traces were thus just referred to divicine and isouramil ions generated in gas phase from vicine and convicine ions.

**Figure 4:**
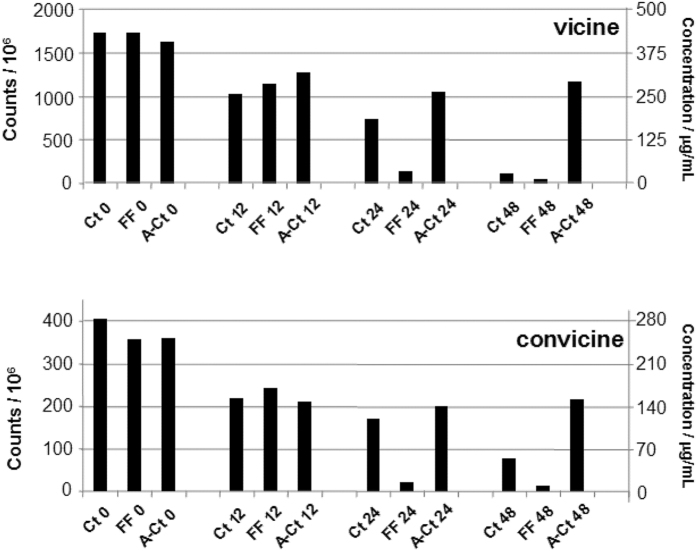
Comparison between ESI-MS XIC peak areas obtained for vicine and convicine after the LC-ESI-MS analyses of the acid extracts of different faba bean flour doughs, incubated at 30 °C for 0, 12, 24 and 48 h: FF, fermented dough, inoculated with *Lactobacillus plantarum* DPPMAB24W (7.30 log cfu/g initial cell density); Ct, not-inoculated control dough; A-Ct, not inoculated and chemically acidified dough, added with antibiotics (0.1% wt/vol, cycloheximide, and 0.1% wt/vol chloramphenicol). Concentrations estimated for vicine, through a calibration performed using standard vicine, and for convicine, based on an indirect evaluation of its ESI-MS sensitivity factor (see text for details), are also reported.

In the further step of this study the attention was then focused on vicine and convicine still present in the flour extracts. In particular, the areas of peaks detected in the corresponding XIC traces (*m/z* 305.3 and 306.3) were used to compare the different doughs on a quantitative basis (Fig. 4). Indeed, those areas were expected to be proportional to the concentrations of the two compounds in each sample, provided that between-analysis fluctuations of the ESI-MS response were not significant. This assumption was verified experimentally, by comparing the XIC peak areas obtained from LC-ESI-MS analyses of the 1 mM solution of standard vicine, performed three times for each sample analysis. In fact, vicine MS response fluctuations were not higher than 10%. In order to estimate vicine residual concentration in each flour extract from its XIC peak area a calibration was performed on standard vicine solutions prepared in 5% perchloric acid (i.e., the same solvent of the flour extracts) in the concentration range 10–100 μg/mL. Concentrations values estimated for vicine from its calibration line were then indicated, along with XIC peak areas, in the relevant plot of Fig. 4. The described calibration was not possible for convicine, due to the lack of a standard. However, starting from the convicine/vicine XIC peak area ratio obtained during the LC-ESI-MS analysis of the extract of unprocessed faba bean flour (see Fig. 3) and from the estimated convicine/vicine concentration ratio obtained for a flour of the same batch during a previous study^18^, the ESI-MS sensitivity factor, i.e., the factor correlating XIC peak area and concentration of convicine could be also estimated and found to correspond to the 35% of that of vicine. This result is not surprising, since convicine has one NH_2_ group less than vicine and the NH_2_ group is a key structural feature to promote the generation of [M+H]^+^ ions during the ESI process. Estimates of convicine concentrations in the analyzed flour extracts, based on the estimated ESI-MS sensitivity factor, are also reported in the relevant plot of Fig. 4.

As shown in the figure, the differences in vicine and convicine XIC peak areas related to samples analyzed before incubation were not significantly (P < 0.05) higher than those observed during replicated analysis of the same standard solution of vicine, in relative terms. A decrease was found after the first 12 hours of incubation but it was almost comparable for the three types of dough, as if a chemical degradation of vicine and convicine had occurred in the first stage of incubation. Differences between the three types of doughs became apparent after 12 hours. Indeed, the degradation of the two compounds was clearly more effective in FF, in which the presence of vicine and convicine was barely detectable after 48 hours of fermentation. Compared to that reached after 12 hours, no further degradation of the two compounds was observed for A-Ct, while Ct was characterized by a progressive decrease of their MS responses (i.e. of their concentrations). Nevertheless, the values obtained after 48 hours of incubation were higher than those found for FF, especially for convicine.

### Blood hemolysis

**Figure 5:**
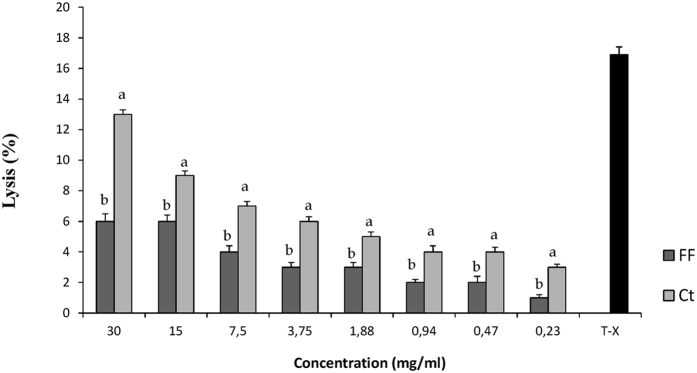
Hemolysis of human red blood cells treated with different concentration of the extracts obtained by the method of Marquardt and Fröhlich (1981) from faba bean flour doughs (dough yield of 200) incubated at 30 °C for 48 h. FF, fermented dough, inoculated with *Lactobacillus plantarum* DPPMAB24W (7.30 log cfu/g initial cell density); Ct, spontaneously fermented control dough. ^a–c^For each concentration, values with different superscript letters differ significantly (*P* < *0.05*). Triton-X (T-X) (o.1% wt/vol) was used as positive control.

The determination of human blood hemolysis was carried out using the freeze-dried extracts from FF 48 and Ct 48 faba bean flour doughs, obtained by the method proposed by Marquardt and Fröhlich^19^. Triton X-100, used as positive control (0.1% wt/vol), caused the hemolysis of 16.9% of red blood cells (see Fig. 5). The effect of the extracts obtained from the two types of faba bean doughs under comparison resulted clearly dependent on the concentration tested. Moreover, the degree of red blood cells hemolysis caused by the Ct extracts was significantly (*P* < *0.05*) higher than FF at any dilution, thus confirming the results shown in Fig. 4, i.e. the presence of much lower concentrations of vicine and convicine in the FF samples. In particular, the degree of hemolysis caused by the FF extract was 54 and 40% lower than Ct, respectively, when the extract was used at concentrations of 30 and 15 mg/mL (Fig. 5).

## Discussion

The importance of legumes in the agricultural system, and the transition to a more plant-based diet were discussed and promoted by several studies, as being environmentally more sustainable^3^,^20^. Faba bean is considered a good substitute for meat-based products in the human diet and could replace soy proteins in both feed and food products, reducing the reliance on imported soymeal. Its nutritional profile and the richness and diversity of bioactive compounds highlight the role of faba beans in maintaining human health and disease prevention^2^,^12^. As a consequence, researchers emphasize the importance of technological innovations able to achieve faba bean products having optimum nutritional value and consumer acceptability^12^.

The pyrimidine glycosides vicine and convicine are toxic to individuals carrying a genetic deficiency of the G6PD^2^ but also have an anti-nutritional effect in the diet of monogastric animals^21^,^22^, limiting its use also in the feed industry. Up to date, the most common industrial and domestic food processing procedures mostly affected the native β-glucosidase activity of faba bean and reduced the content of vicine and convicine^2^,^12^. However, the conversion of vicine and convicine into their toxic derivatives is possible via β-glucosidases of the small intestine, the same involved in the absorption and metabolism of dietary (iso)flavonoid glucosides^23^. Therefore, the complete hydrolysis of the pyrimidine glycosides before ingestion is a necessary pre-requisite to avoid potentially adverse reaction in genetically predisposed individuals.

In our previous study, fermentation with *L. plantarum* DPPMAB24W successfully reduced the glucosides while at the same time enhancing the nutritional properties of faba bean flour^18^. In this further investigation, the effectiveness of the fermentation system on degrading the aglycones derivative compounds was monitored throughout bioprocessing. The effects of fermentation with *L. plantarum* were compared to control treatments (spontaneous fermentation and chemically acidified control with antibiotics), assessing the efficacy of the method. *L. plantarum* is a ubiquitous species, found in several food ecosystems due to its versatile metabolism and the capacity of large adaptation to different environments. The kinetics of growth and acidification of the starter during faba bean flour fermentation were previously monitored, assessing the strain performance in the same fermentation conditions here adopted. Fermentation of faba bean flour with selected strain of *L. plantarum* was characterized by a relatively long adaptation phase but was able to dominate on the spontaneous microbiota^18^. Analogous behavior was observed in this study, in which controlled fermentation with the strain *L. plantarum* DPPMAB24W allowed to achieve a lower pH more rapidly, in comparison with spontaneous fermentation.

In a preliminary phase of the research, an efficient chromatographic method for the separation of vicine from convicine was developed in order to achieve a reliable identification of the two compounds in the dough extracts. For this purpose, the acid extract obtained from the untreated faba bean flour^19^ containing both vicine and convicine, was analyzed by LC-ESI-MS and MS/MS. Since, to the best of our knowledge, no convicine standard is available commercially at the moment, this extract represented a fundamental sample to assess the best conditions for the HPLC separation of vicine and convicine and, additionally, to obtain the MS/MS fragmentation pattern for the latter, as previously done using a vicine commercial standard. Vicine and convicine can be classified as polar compounds, and an excellent separation between vicine and convicine was accomplished during the isocratic step of the elution LC gradient, characterized by a very polar mobile phase.

Previously, it was shown that the effectiveness of β-glucosidase is depending on enzyme concentration, time, temperature and pH conditions, indicating the importance of acidification^9^. In the condition of our study, no difference in the β-glucosidase activity was observed before and after addition of lactic acid and subsequent incubation in the chemically acidified dough, nonetheless a slight decrease of vicine and convicine occurred in the first 12 h of incubation. The differences between the treatments became visible after the first 24 h, and in particular between controlled and spontaneous fermentation. A more intense and faster degradation of the compounds was achieved only when selected lactic acid bacteria were used to inoculate the flour. In this treatment, the β-glucosidase activity was ca. 2.7-times higher than spontaneously fermented faba bean flour dough. This result is not surprising, since β-glucosidase is widespread within lactic acid bacteria, but the level of expression is largely dependent on the strain^24^. In another study, *L. plantarum* DPPMAB24W showed the highest β-glucosidase activity among more than one hundred strains of lactic acid bacteria isolated from different food ecosystems belonging to *L. plantarum*, *Lactobacillus alimentarius*, *Lactobacillus brevis*, *Lactobacillus casei*, *L. casei* subsp. *pseudoplantarum, Lactobacillus delbruecki* subsp. *bulgaricus*, *Lactobacillus helveticus*, *Lactobacillus hilgardii*, *Lactobacillus paralimentarius*, *Lactobacillus paraplantarum*, *Lactobacillus pentosus*, *Lactobacillus sanfranciscensis*, *Lactococcus lactis* subsp. *lactis*, *Lactobacillus parabuckneri*, *Lactobacillus fermentum*, *Lactobacillus gasseri*, *Lactobacillus parabuckneri*, and *Weissella cibaria*^25^. In particular, it was reported that ca. 40% of the strains considered did not exhibit activity, and only few strains were characterized by an activity higher than 0.055 U (median value was 0.030 U). *L. plantarum* DPPMAB24W was the only strain showing an activity higher than 0.160 U, corresponding to 0.202 U^25^. This value almost approaches that found at the end of fermentation in fermented faba bean flour.

When *L. plantarum* DPPMAB24W was used as starter for faba bean flour fermentation, more than 90 and 95% of the initial concentration of vicine and convicine, respectively, were degraded, while only a partial degradation of the two compounds (especially convicine) was found after 24 and 48 h of spontaneous fermentation. In previous studies, vicine and convicine were almost completely removed from whole seeds of faba bean after continuous flow soaking for several hours, and reduction of the glycosides ranging from ca. 20 up to 40% were observed by domestic techniques such as boiling, roasting, and frying (as reviewed by Multari *et al*.^12^), even though information about effective toxicity was missing. The removal of vicine and convicine reached 93 and 100% respectively after seeds germination, overnight storage and further treatment with H_2_O_2_. Their toxicity index however, as referring to glutathione (GSH) destruction, remained still very high^15^. Generally, detoxification treatments on faba bean flours have been more successful compared to whole seed or parts of it. In particular, almost complete removal of the aglycones and low toxicity index were observed after treatments with highly acidic buffers (pH 3.5) plus H_2_O_2,_ implying the importance of acidic conditions on effective detoxification^15^. However, the treatments already proposed are difficult to be applied on a large-scale and often not applicable for food and feed production. Moreover, no information on the detoxification mechanism were reported. In this work, aiming at the identification of the aglycones divicine and isouramil in doughs through LC-ESI-MS, a procedure to distinguish the forms generated from the spontaneous fragmentation of vicine and convicine inside the ESI source and/or the ion optics of the LCQ spectrometer from those deriving from the degradation of the compounds during the dough incubation, was developed and confirmed by the analysis of the enzymatic hydrolysis of the vicine/convicine extracts. Irrespective of the treatment used, the extracts obtained from fermented faba bean flour doughs revealed that, once generated, the aglycones of vicine and convicine are not stable under the conditions adopted. It is likely that the oxygen dissolved into doughs leads to their further, rapid degradation, with the final rupture of the pyrimidine ring, as previously hypothesized by Chevion *et al*.^26^.

This evidence represents a very encouraging result in terms of safety of the fermented faba bean flour for individuals suffering from favism or related pathologies.

The favic crisis has been defined as a paradigm for oxidant damage to the human red blood cells^27^. The acute hemolysis caused by faba bean ingestion in G6PD-deficient individuals shows strong analogies to hemolytic crises caused by different oxidant chemicals. In the G6PD deficient red blood cells no regeneration of GSH occurs even after several hours of incubation in presence of glucose^8^. In normal red blood cells, oxidized GSH is rapidly regenerated by a metabolic cycle of which G6PD is an essential component. In fact, experiments on normal volunteers have shown that ingestion of 500 g of commercially available faba beans elicited a 30–40% drop in the GSH level within 3 h from ingestion^2^. However, in this case, the original GSH is quickly regenerated without causing negative effects. The favic crisis brings a massive and rapid senescence of a large number of red blood cells in which these senescent red blood cells are removed similarly as in normal red blood cells senescence, but the rate of the process can be magnified up to 15-fold^12^.

In the hemolytic assay performed using normal red blood cells, the hemolysis caused by the extract of lactic acid bacteria fermented faba bean flour was less than half compared to the spontaneously fermented control, at the highest concentration tested (30 mg/ml). This result indicate that detoxification of faba bean is possible with mild and cost effective technologies, such as a bioprocessing with selected lactic acid bacteria. The findings of this study open up to new perspective for implementing faba bean uses in the food industry, as strongly suggested by the scientific community and international governmental and non-governmental organizations, with very positive repercussion on global markets, human diet, and environment sustainability.

## Materials and Methods

### Faba bean flour composition

Six batches of commercial Italian faba bean (*Vicia faba* major, harvest year 2014) flours, obtained from the stone-milling of the dehulled seeds, were purchased from CerealVeneta (San Martino di Lupari, PD, Italy) and pooled before the use. Moisture was determined by oven drying the flour at 130 °C for 1 hour according to method 44–15.02^28^. Total protein concentration was analyzed according to the method 46-11A^28^ with Kjeldahl autoanalyser (Foss Tecator Ab, Höganäs, Sweden). Lipids were determined using Soxhlet extractor (Büchi B-811, Labortechnik AG, Flawil, Switzerland). Ash content was determined with gravimetric method using Naber N11 ash oven (Nabertherm, Germany). Total carbohydrates were calculated as the difference [100 − (proteins + lipids + ash + moisture)]. Total dietary fibre was analyzed with the enzymatic-gravimetric method 985.29^29^.

### Bacterial strain, growth conditions and β-glucosidase activity

*Lactobacillus plantarum* DPPMAB24W (also known as VTT E-133328) from the Culture Collection of the Department of Soil, Plant, and Food Science (University of Bari “Aldo Moro”, Italy) whose selection was based on the β-glucosidase activity^18^,^25^ was used as starter for faba bean flour fermentation. The lactic acid bacteria strain was routinely propagated at 30 °C in MRS broth (Oxoid, Basingstoke, Hampshire, England).

For the determination of the β-glucosidase activity of flour and doughs, water/soluble extracts (WSE) were prepared as described by Weiss *et al*.^30^. The β-glucosidase activity was measured in terms of *p*-nitrophenol released from *p*-nitrophenyl-β-D-glucopyranoside (*p*NPG) substrate (Sigma-Aldrich, Milan, Italy) as described in Di Cagno *et al*.^25^. One unit (U) of enzyme activity was defined as the amount of β-glucosidase that released 1 μmol of *p*-nitrophenol from the substrate *p*NPG per milliliter per min under the assay conditions. Calibration curve for *p*-nitrophenol (Sigma) was obtained using concentrations in the range of 0.05–2.00 mM.

### Faba bean flour treatments

When used for fermentation, *L. plantarum* DPPMAB24W was cultivated (at 30 °C in MRS broth) until the late exponential phase of growth was reached (ca. 10 h). Cells were recovered by centrifugation (10,000 × g, 10 min), successively washed twice in 0.05 M phosphate buffer, pH 7.0, and re-suspended in the tap water (ca. 10% of the initial volume of the culture) used for the preparation of the dough.

Faba bean flour was mixed with water in a ratio of 50:50 (wt:vol), using a IM 5–8 high-speed mixer at 60 *rpm* for 5 min (Mecnosud, Flumeri, Italy).

Three different doughs were prepared: a fermented dough (FF), inoculated with the starter (7.30 log cfu/g initial cell density) and incubated at 30 °C for 48 h, and two not-inoculated doughs, incubated in the same conditions and used as controls: Ct, representing a spontaneous fermentation; A-Ct, added with antibiotics (0.1% wt/vol cycloheximide and 0.1% wt/vol chloramphenicol) and acidified to pH 4.5 with lactic acid. Aliquots of all the doughs were taken before incubation (T0), and after 2 (T2), 12 (T12), 24 (T24), and 48 h (T48) of incubation and used for further analyses.

### Microbiological analysis

Microbiological analysis of the samples were performed at the beginning and end of fermentation as described in Coda *et al*.^18^. Lactic acid bacteria were enumerated using MRS (Oxoid, Basingstoke, Hampshire, UK) agar medium, supplemented with cycloheximide (0.1 g/L). Plates were incubated under anaerobiosis (AnaeroGen and AnaeroJar, Oxoid) at 30 °C for 48 h. Cell density of yeasts and molds were estimated on Yeast Extract Peptone Dextrose Agar (YPD) (Oxoid) medium, supplemented with chloramphenicol (0.15 g/L), at 30 °C for 72 h. Total mesophilic aerobic bacteria were determined on Plate Count Agar (PCA, Oxoid) at 30 °C for 48 h and total enterobacteria were determined on Violet Red Bile Glucose Agar (VRBGA, Oxoid) at 37 °C for 24 h. Until the chemical analyses, doughs were immediately freezed at −20 °C at the end of incubation.

### Determination of pH and organic acids

The values of pH and Total Titratable Acidity (TTA) were determined as previously described by Rizzello *et al*.^31^. TTA was expressed as the amount (mL) of 0.1 M NaOH needed to reach a pH value of 8.3. WSE of faba bean flour doughs were prepared according to Weiss *et al*.^30^ and used to analyze organic acids by High Performance Liquid Chromatography (HPLC), as described in Coda *et al*.^32^.

### Extraction of vicine and convicine

The extraction of vicine, convicine and their aglycones was performed with the method proposed by Marquardt and Fröhlich^19^ with some modifications. Fifty milliliters of perchloric acid (5% wt/vol) were added to 5 g of the faba bean flour doughs. Samples were homogenized for 5 min at 4 °C. The extract was centrifuged and filtered through a 0.45 μm filter (Millipore Co., Bedford, MA) to remove suspended material before the analyses based on LC coupled to mass spectrometry (MS). For hemolysis determination the extracts were freeze-dried to remove the solvent.

The set-up of the LC-MS method could be applied experimentally only for vicine (purchased from Sigma), due to the lack of a commercial convicine standard. In particular, 1 mL of a 1 mM solution of vicine prepared in 50 mM phosphate buffer at pH 5, preliminarily flushed with nitrogen for 10 min, was subjected to enzymatic hydrolysis by adding 5 mg of almond β-glucosidase (Sigma) and incubating at room temperature, in accordance with the experimental conditions reported by Pedersen, Musci and Rotilio^33^. Aliquots of the mixture, resulting from flour extracts after enzymatic hydrolysis of the vicine/convicine (E-Ct) were withdrawn, at 30 min intervals, over a 2 h time of incubation and analyzed by the LC-ESI-MS method described below.

### Liquid chromatography-mass spectrometry (LC-MS) analysis

A LC-MS system consisting in a *1525 micro* liquid chromatograph (Waters, Milford, MA, USA), a single wavelength UV-VIS detector Agilent 1100 (Agilent, Santa Clara, CA, USA) and a LCQ 3D-ion trap mass spectrometer (ThermoElectron, San Jose, CA, USA) equipped with an ElectroSpray Ionization (ESI) interface was used for the separation and analysis of standard vicine, faba bean flour extracts (naturally containing vicine and convicine), E-Ct (the mixtures resulting from the enzymatic hydrolysis of flour extract) and, finally, of extracts of faba bean flour doughs (FF, Ct, and A-Ct). In particular, a reversed phase liquid chromatographic separation was accomplished, at a 0.2 mL/min flow, using a Supelcosil LC-18 column (250 × 2.1 mm, packing particles diameter: 5 μm), containing an octadecylic (C18) stationary phase. The following binary elution gradient, based on water (A) and acetonitrile (B), both containing 0.1% (v/v) of formic acid, was adopted for the separation: 0–3 min) isocratic at 0% B; 3–4 min) from 0 to 10% B; 4–14 min) isocratic at 10% B; 14–19 min) from 10 to 90% B; 19–29 min) isocratic at 90% B; 29–34 min) return to 0% B. The program was followed by a 16-min reconditioning step, thus the entire run length was 50 min. A 6-way Rheodyne valve, equipped with a 20 μL loop, was adopted for manual sample injections, whereas the liquid chromatograph was controlled by the MassLynx 4.0 software (Waters, Milford, MA, USA). The column effluent was first transferred to the UV-VIS detector, operated at 280 nm, in order to detect both vicine and convicine and their aglycones. The detector effluent was then introduced into the LCQ mass spectrometer through its ESI interface.

The LCQ spectrometer, controlled by the Xcalibur software (ThermoElectron, San Jose, CA), was operated in positive ion mode, since vicine, convicine and their aglycones could be detected as mono-protonated ions ([M+H]^+^) at the low pH (about 4) recreated in the chromatographic mobile phase through formic acid addition. The values adopted for the ESI interface and ion optics parameters of the LCQ spectrometer and the single (MS) and tandem (MS/MS) mass spectrometry acquisitions performed during the present investigation have been described in detail in Section S1 of the Supplementary Information.

### Human blood hemolysis

The determination of the human blood hemolysis was carried out by Cyprotex laboratories (Watertown, MA, USA). The analysis was performed with appropriate local health regulations and ethical approval. In particular, human blood was provided under the federal guidelines for sample collection by the Research Blood Components (Brighton, MA, USA), following the American Association of Blood Banks guidelines for drawing donors. The experimental protocol was approved by the U.S. Food and Drug Administration (FDA). The freeze-dried extracts from faba bean flour doughs were used at concentrations ranging from 0.23 to 30 mg/mL, dissolved in saline at 300 mg/mL (wt/vol) and sonicated for 15 minutes. Afterwards, the mixtures were centrifuged (12000× *g*, 10 min) and the supernatant was diluted for the assay. Human, heparinized commercial blood was stored at room temperature and used within 8 h of collection. The dilutions of each supernatant were prepared in saline and then diluted into aliquots of blood. Triton X-100 (Sigma) was used as positive control. The blood was incubated at 37 °C for 4 h with gentle shaking and then centrifuged to separate the cells from the plasma. An aliquot of plasma was diluted with Drabkin’s reagent (leading to the conversion of hemoglobin to cyanmethemoglobin) and the OD540 was measured. A calibration curve prepared by dilution of non-centrifuged blood was used to determine the degree of hemolysis. Three samples for each extract, obtained by independent experiments, were twice analyzed and the means of the data were statistically treated (as described below) to assess the significant differences against the control values.

### Statistical analysis

Data were subjected to one-way ANOVA; pair-comparison of treatment means was achieved by Tukey’s procedure at P < 0.05, using the statistical software Statistica 7.0 (Statsoft) for Windows.

## Additional Information

**How to cite this article**: Rizzello, C. G. *et al*. Degradation of vicine, convicine and their aglycones during fermentation of faba bean flour. *Sci. Rep.* **6**, 32452; doi: 10.1038/srep32452 (2016).

## Supplementary Material

Supplementary Information

## Figures and Tables

**Table 1:** Chemical and microbiological characteristics of faba bean doughs incubated at 30 °C for 48 h.

	FF	Ct	A-Ct
pH	4.52^b^	5.11^a^	4.43^b^
TTA	16.10^a^	12.70^b^	15.79^a^
Lactic acid (mmol/kg of dough)	105^b^	41^c^	118^a,^*
Acetic acid (mmol/kg of dough)	18^a^	12^b^	n.d.
Lactic acid bacteria (Log cfu/g of dough)	9.69^a^	6.70^b^	5.70^c^
Yeasts (Log cfu/g of dough)	1.51^b^	2.83^a^	2.70^a^
β-glucosidase activity (U)	0.198^a^	0.072^b^	0.040^c^

FF, fermented dough, inoculated with *Lactobacillus plantarum* DPPMAB24W (7.30 log cfu/ml initial cell density); Ct, spontaneously fermented control dough; A-Ct, not inoculated and chemically acidified dough, added with antibiotics (0.1% wt/vol, cycloheximide, and 0.1% wt/vol chloramphenicol). The data are the means of three independent experiments. ^a–c^Values in the same row with different superscript letters differ significantly (*P < 0.05*).

^*^Lactic acid was added for the chemical acidification.
