# Supplementary material for: Degradation of vicine, convicine and their aglycones during fermentation of faba bean flour
Source: Sci Rep. 2016 Aug 31;6:32452. doi: 10.1038/srep32452 (PMC5006014; doi:10.1038/srep32452)
Supplement: Supplementary Information [file srep32452-s1.doc]

**Degradation of vicine, convicine and their aglycones during fermentation of faba bean flour**

*Carlo Giuseppe Rizzello, Ilario Losito, Laura Facchini, Kati Katina, Francesco Palmisano, Marco Gobbetti, Rossana Coda*

**Supplementary information**

**S1. Values adopted for the main parameters of the ESI interface and ion optics of the LCQ spectrometer and details on the mass spectrometric acquisitions performed during LC-MS analyses**

The following values were adopted for the ESI interface and ion optics main parameters: sheath gas flow rate, 60 (a.u.); auxiliary gas flow rate, 0 (a.u.); spray voltage, +6 kV; capillary temperature, 190°C; capillary voltage, 15 V; tube lens offset, 10 V; octapole 1 offset, −2.5 V; lens voltage, −16 V; octapole 2 offset, −5.5 V; octapole RF amplitude, 400 V_p‑p_.

Different MS acquisitions were usually performed during each run, namely a MS *full scan* in the 50–2000 *m/z* range and a series of tandem mass spectrometry (MS/MS) acquisitions. The latter were performed by isolating each time, as a precursor ion, only the main isotopologue of the [M+H]^+^ ion of vicine, convicine, divicine or isouramil (i.e., the ion arising from the molecular structure including only the most abundant isotope for each constituting atom), using a 1 *m/z* units-wide window centered on the *m/z* ratios 305.3, 306.3, 143.2 and 144.2, respectively. It is worth noting that the isolation window did not include the entire isotopic pattern of each of the four ions, since, in case of partial co-elution from the C18 column, interferences between the fragmentation patterns related to the second isotopologues of vicine (*m/z* 306.3) and divicine (144.2) ions and to the first isotopologues of convicine and isouramil ions, having the same *m/z* ratios, respectively, would have occurred. In all cases, fragmentation was promoted through Collisional Induced Dissociation (CID) inside the 3D-ion trap of the LCQ spectrometer, at a collisional energy equal to 35% of the maximum value (in turn corresponding to a 5 V_p-p_ RF voltage applied to the end caps of the ion trap). The upper *m/z* limit for each MS/MS spectrum was always set as 10 *m/z* units higher than the precursor ion *m/z* value, whereas the lower limit (*Low Mass Cut Off*) was automatically determined by the instrument.

**S2. Distinction of divicine and isouramil generated in solution from those arising, respectively, from gas-phase spontaneous degradation of vicine and convicine during MS analysis.**

The evaluation was preliminarily made on vicine, due to the lack of a commercial convicine standard. In particular, 1 mL of a 1 mM solution of vicine prepared in 50 mM phosphate buffer at pH 5, preliminarily flushed with nitrogen for 10 min (to avoid the eventual oxidation of generated divicine, or of vicine itself, by atmospheric oxygen), was subjected to enzymatic hydrolysis by adding 5 mg of almond β-glucosidase (Sigma Aldrich, Milan, Italy) and incubating at room temperature, in accordance with the experimental conditions reported by Pedersen, Musci and Rotilio (1988). Aliquots of the mixture were withdrawn, at 30 min intervals, over a 2 h time of incubation and analyzed by the LC-ESI-MS method developed before. As a result, a progressive decrease of the peak related to vicine was observed in the XIC trace obtained for the *m/z* 305.3 ion and it became virtually undetectable after 2 hours of reaction. On the other hand, a peak whose intensity increased with reaction time was found in the XIC trace obtained for the *m/z* ratio corresponding to divicine (*m/z* 143.1), thus confirming that the hydrolysis reaction had occurred.

Since, as pointed out before, vicine is also able to generate divicine in gas phase during ESI-MS measurements, as a result of spontaneous fragmentation inside the ESI source and/or the ion optics of the LCQ spectrometer, a comparison was made between the XIC traces related to the *m/z* ratio 143.1 obtained before starting the enzymatic hydrolysis reaction (when only vicine was present, thus the signal could be due only to gas phase-generated divicine) and after 2 hours (when only divicine generated in solution was present) and is shown in Figure 1S.

As apparent from Figure 1S, a larger (compared to that obtained for standard vicine) and composite peak, likely arising from the partial co-elution of two single peaks with different relative intensity, was found after 2 hours of enzymatic reaction. This result could be due to the presence of divicine in the two tautomeric forms shown in Figure 1, likely having a slightly different retention time. Unfortunately, as clearly evidenced in Figure 1S, the narrow peak related to standard vicine and the broad peak related to divicine generated in solution were co-eluting and, although several attempts were made, they could not be separated, maybe due to the fact that the relevant part, in terms of interaction with the C18 stationary phase, of the molecular structures of vicine and divicine, i.e. the pyrimidine ring, is identical. Nonetheless, a careful, parallel monitoring of ion currents related to *m/z* ratios 305.3 and 143.1 offered the possibility of understanding the real nature of the signal at *m/z* 143.1 (divicine generated in solution vs divicine corresponding to the product of vicine spontaneous fragmentation inside the mass spectrometer) at any time of the enzymatic reaction and, subsequently, in real samples potentially containing both vicine and divicine, like the extracts of faba bean flour doughs. As shown in Figure 2S, this check indicated that no significant concentration of divicine was present in the doughs. A similar approach was adopted to monitor, in the real samples, the *m/z* 306.3 and 144.1 ions, related, respectively, to convicine and to isouramil (again, generated in solution during fermentation or resulting from the fragmentation of convicine inside the mass spectrometer). By analogy with the chromatographic behavior of vicine/divicine, a co-elution between convicine and chemically generated isouramil, with the peak due to the latter being wider than that related to convicine, due to tautomerism, was expected if isouramil arising from in solution degradation of convicine was present. However, as shown by some examples reported in Figure 3S, no peak broadening was ever observed in the XIC traces referred to the *m/z* ratio 144.1, thus suggesting that isouramil, like divicine, never reached significant concentrations in the faba bean flour doughs.

**
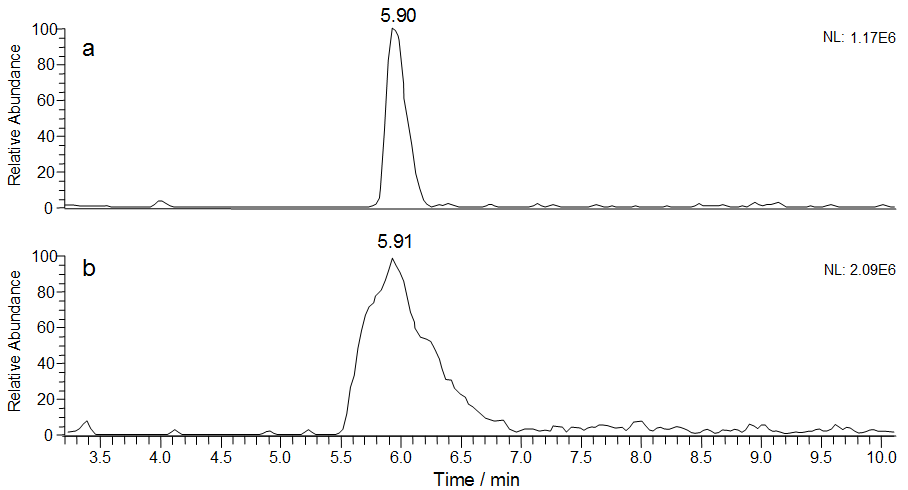
**

**Figure 1S**. Comparison between the LC-ESI-MS eXtracted Ion Current (XIC) chromatograms obtained for the *m/z* range 143.1±0.5, corresponding to the main isotopologue of the [M+H]^+^ ion of divicine, for the following sample: a) 1 mM vicine standard in 50 mM PBS (pH 5); b) the same solution after 2 h of hydrolysis reaction catalyzed by almond β-glucosidase. Note that the peak shown in panel b is due exclusively to divicine generated in solution, since the residual vicine concentration after 2 h of reaction was negligible.

**
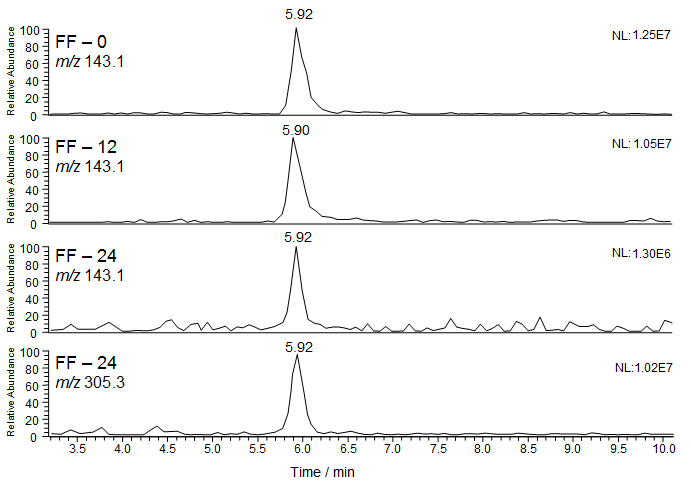
**

**Figure 2S**. Comparison between the LC-ESI-MS eXtracted Ion Current (XIC) chromatograms obtained for the *m/z* ranges 143.1±0.5 or 305.3±0.5, corresponding, respectively, to the main isotopologues of the [M+H]^+^ ions of divicine and vicine, referred to the FF samples analyzed before and after 12 and 24 hour of fermentation. The shape similarity clearly observed for peaks referred to divicine and vicine in the FF-24 sample was found also in all the other flour extracts, including those belonging to the Ct and A-Ct series. This result confirmed that the detected divicine was generated only upon gas-phase fragmentation of vicine during the ESI-MS analysis, thus the aglycone never reached appreciable concentrations in the flour doughs.

**
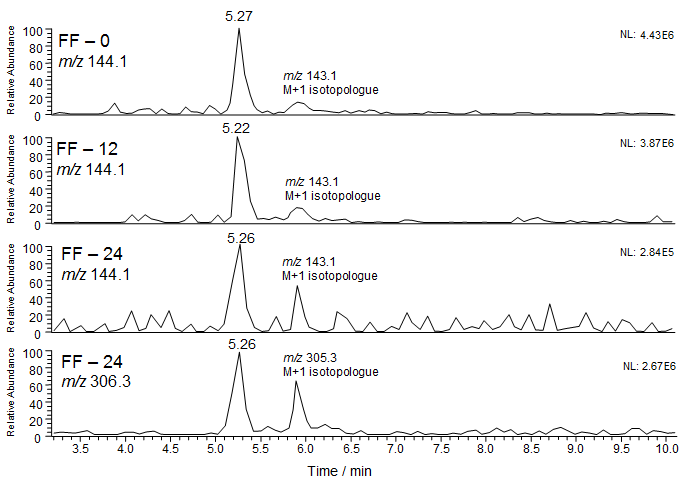
**

**Figure 3S**. Comparison between the LC-ESI-MS eXtracted Ion Current (XIC) chromatograms obtained for the *m/z* ranges 144.1±0.5 or 306.3±0.5, corresponding, respectively, to the main isotopologues of the [M+H]^+^ ions of convicine and isouramil, referred to the FF samples analyzed before and after 12 and 24 hour of fermentation. The shape similarity clearly observed for peaks referred to convicine and isouramil in the FF-24 sample was found also in all the other flour extracts, including those belonging to the Ct and A-Ct series. This result confirmed that the detected isouramil was generated only upon gas-phase fragmentation of convicine during the ESI-MS analysis, thus the aglycone never reached appreciable concentrations in the flour doughs.
